# Long-Term Survival of Patients With Glioblastoma of the Pineal Gland: A ChatGPT-Assisted, Updated Case of a Multimodal Treatment Strategy Resulting in Extremely Long Overall Survival at a Site With Historically Poor Outcomes

**DOI:** 10.7759/cureus.36590

**Published:** 2023-03-23

**Authors:** Andrew R Cunningham, Hayley E Behm, Andrew Ju, Matthew S Peach

**Affiliations:** 1 Department of Radiation Oncology, Brody School of Medicine, East Carolina University, Greenville, USA

**Keywords:** radiotherapy, bevacizumab, temozolomide, leptomeningeal spread, ventricular radiation, chatgpt, pineal gland, glioblastoma

## Abstract

We present an updated case report of a patient with glioblastoma isolated to the pineal gland with an overall survival greater than five years and no progression of focal central nervous system (CNS) deficits since initial presentation. The patient underwent radiotherapy up to 60 Gy with concurrent and adjuvant temozolomide with the use of non-standard treatment volumes that included the ventricular system. The utilization of ventricular irradiation as well as the addition of bevacizumab at disease recurrence may have encouraged this unusually long survival by preventing/delaying leptomeningeal spread. We also present an updated review of the literature, which shows a median survival of six months, reinforcing the patients atypical disease trajectory. Finally, we utilize OpenAI’s language model ChatGPT to aid in synthesizing this manuscript. In doing so, we demonstrate that ChatGPT is apt at creating concise summaries of relevant literature and topic subjects, however its output is often repetitive with similar sentence/paragraph structure, less than ideal grammar and poor syntax requiring editing. Thus, in its current iteration, ChatGPT is a helpful aid that cuts down on the time spent in data acquisition and processing but is not a replacement for human input in the creation of quality medical literature.

## Introduction

Glioblastoma of the pineal region is an uncommon location for what is the most common primary central nervous system (CNS) malignancy, with 40 cases reported in the literature. Despite advances in cancer treatment, overall survival (OS) for patients with pineal gland glioblastoma is often in the span of months [1], making this site a significant clinical challenge compared to typical glioblastomas, where the median OS is 1.5 years [2]. The pineal gland is a small, pinecone-shaped structure located in the center of the brain. It plays an essential role in regulating circadian rhythms and secretes melatonin, a hormone that helps regulate sleep-wake cycles. Pineal gland tumors are rare, accounting for less than 1% of all brain tumors, with an extremely small fraction consisting of glioblastoma.

Glioblastoma of the pineal region presents a unique diagnostic and therapeutic challenge due to its location. In being bounded by the third ventricle and quadrigeminal cistern, disease can disseminate along the ventricular/cistern surfaces and be broadcasted through the CSF fluid, settling along the neuraxis. Additionally, the pineal gland is adjacent to critical structures such as the brainstem and optic structures. Treatment options for pineal gland glioblastoma typically involve a combination of surgery, radiation therapy (RT), and chemotherapy. However, the optimal treatment approach for this rare and aggressive site remains unknown.

This case study elaborates on the clinical presentation and treatment course of a patient with pineal gland glioblastoma with an over five-year overall survival, which may not be limited by disease progression. We provide an updated literature review that reinforces the exceptional survival of this patient. To demonstrate the capabilities of language-model artificial intelligence in aiding medical writing, we utilized the assistance of ChatGTP in developing this case report and literature review. By sharing this case, we hope to contribute to the growing body of knowledge on this rare disease site and help inform future treatment decisions for patients with pineal gland glioblastoma.

## Case presentation

Case background and update

**Figure 1:**
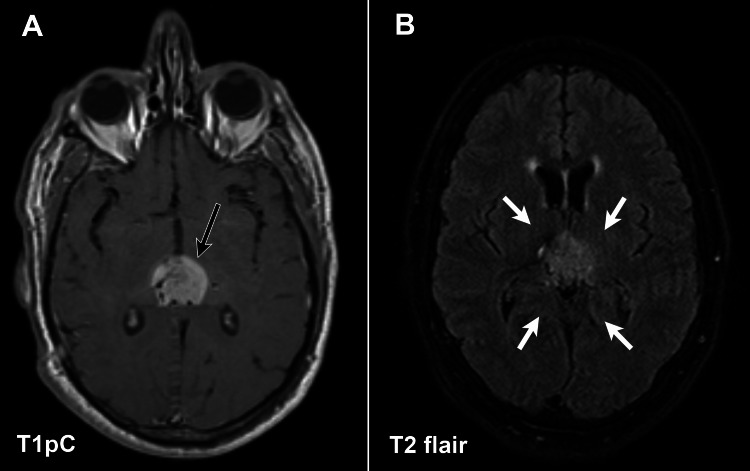
T1 post-contrast (A) and T2 flair (B) MRI at the initial presentation of a pineal glioblastoma. There is an approximate 25-mm enhancing pineal mass on T1 post-contrast imaging (black arrow), with no evidence of edema to the parenchyma highlighted by the white arrows that surround the lesion on T2 flair MRI.

We previously published the case of a 64-year-old man with no significant medical history other than gastroesophageal reflux disease (GERD) who presented with vertical diplopia, headaches, and insomnia, where the neurological exam found right cranial nerve IV palsy and gait difficulties [1]. CT imaging revealed a hyperdense pineal mass, with biopsy demonstrating a glioblastoma histologically (atypical cells with giant nuclei, seven mitoses per three high-powered fields, multiple microvascular foci, pseudopalisading necrosis present) and molecularly (isocitrate dehydrogenase 1/2 wildtype with O-6-methylguanine-DNA methyltransferase (MGMT) promoter hypermethylation). The enhancing mass (Figure 1A) was approximately 25 mm in diameter with no significant surrounding edema, as shown in Figure 1B. Initial treatment details from the radiotherapy treatment plan are elaborated upon in our initial report [1]. Briefly, given the access to the ventricular system, a low dose volume of 50 Gy was applied consisting of the gross enhancing disease as well as compartments typically included in whole-ventricle irradiation with a 2 cm margin. This was followed by a cone-down to gross-enhancing disease with a 2 cm margin to 60 Gy delivered in 2 Gy daily fractions. There was no T2-flair signal beyond the enhancing disease; therefore, these sequences had no effect in developing the low dose volume. He experienced the expected increased in fatigue toward the end of radiotherapy and afterward developed temporary alopecia with no other toxicity. The patient continued with adjuvant temozolomide (TMZ) and had no evidence of disease progression or recurrence until the 12th cycle. At 58 weeks post-biopsy, two new lesions were appreciated in the brainstem and right parietal lobe, at which point bevacizumab (7.5 mg/kg every three weeks) was added to TMZ. Given these radiographic findings, the decision was made to continue TMZ for 18 cycles alongside bevacizumab initiation. Overall, 17 cycles of adjuvant TMZ (one cycle at 150 mg/m^2^, the remaining 200 mg/m^2^ first five days of the 28-day cycle) were completed, with the 18th cycle omitted due to limited prescription availability at the time. TMZ was discontinued at 88 weeks post-biopsy, and bevacizumab was discontinued 129 weeks after biopsy. There were no radiographic changes upon follow-up MRI at 146 weeks post-biopsy. During the interim between this MRI and his prior imaging, the patient underwent physical therapy on a regular basis, which lead to improved diplopia and ataxia.

Since this initial report, the patient continued to undergo close clinical and radiographic surveillance with MRIs every two months. At 154 weeks post-biopsy, the patient exhibited T1 hypersensitivity in the pineal region, measuring 19 mm × 15 mm. By 192 weeks post-biopsy, the pineal region had gradually reduced to 12 mm × 10 mm, while there was an increased enhancement to the right superior colliculus and right periatrial white matter. There was also interval development of a small unrelated parenchymal hemorrhage. The pineal region significantly increased in size to 30 mm × 16 mm × 19 mm at week 201 post-biopsy, with continued increase in enhancement of the atrium of the right lateral ventricle.

**Figure 2:**
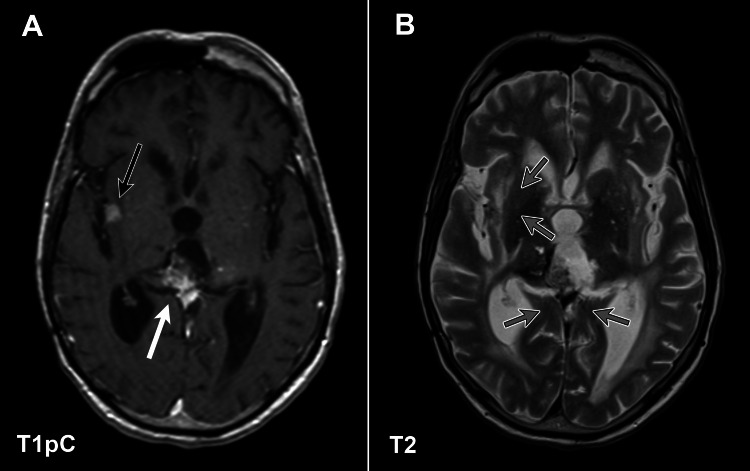
T1 post-contrast (A) and T2 MRI imaging (B) of a recurrent pineal gland glioblastoma with minimal progression at 263 weeks post-biopsy. The pineal gland demonstrates stable post-contrast enhancement (white arrow), while there was mild growth of the enhancing lesion of the right insular cortex to 9 mm (black arrow). The gray arrows demonstrate a lack of edema in the parenchyma surrounding both the primary and right insular cortex lesions on T2 MRI.

At 217 weeks, the pineal region had reduced to 30 mm × 15 mm × 10 mm, while at 234 weeks, a new 3 mm insular region nodule was observed. Both the pineal region and insular region demonstrated interval growth at 256 weeks post-biopsy. During this period, the patient developed decreased appetite with a 15lbs weight loss. He had less mobility at this time as a result of his decrease participation in physical therapy. A final MRI was obtained at 263 weeks post-biopsy, where the enhancing pineal region remained stable in size (Figure 2A), although smaller in volume than at presentation. Figure 2A also delineates the enhancing insular region, which had grown to 9 mm. T2 MRI from this time did not show any evidence of edema in the parenchyma surrounding both lesions as highlighted by the gray arrows in Figure 2B. Mild nodular enhancement was appreciated along the right and left lateral ventricles. There were also several chronic small vessel ischemic changes throughout the white matter and basal ganglia. At this time, he developed a urinary tract infection (UTI) that progressed to urosepsis, and the decision was made to enter hospice care, where he passed away at week 265 post-biopsy. Prior to death, the patient continued to experience diplopia and cognitive deficits that were stable from initial presentation, with no headaches, seizures or paresthesia, and no other changes in vision or focal muscle weakness. Throughout the post-radiation therapy (RT) MRI imaging, there was no radiographic evidence of radionecrosis to the brain parenchyma.

Updated literature review

Since our last review in 2017 [1], there have been four published case studies into glioblastoma of the pineal region with a total of 14 cases. Orrego et al. published a case study with four unique cases of pineal glioblastoma in the Neurosurgery Department at the Instituto Nacional de Enfermedades Neoplasicas between 1994 and 2012 [3]. Case 1 was a 48-year-old female patient diagnosed with a pineal tumor (glioblastoma) who underwent a ventricular peritoneal shunt and subtotal resection. She received adjuvant radiation therapy but died 12 months after surgery. Case 2 was a 50-year-old male patient diagnosed with a pineal tumor (glioblastoma) who underwent a ventricular peritoneal shunt and partial resection. His radiotherapy was discontinued prior to completion, and he died six months after surgery. Case 3 was a 56-year-old male who underwent partial resection of his pineal glioblastoma with a ventricular peritoneal shunt. He received radiation therapy with concurrent TMZ but developed new symptoms nine months later and chose palliative care. He died 29 months after surgery. The final case involved a 25-year-old male patient who was diagnosed with a pineal glioblastoma and underwent a ventricular peritoneal shunt and maximal safe resection. He received radiation therapy with concurrent TMZ but developed a local recurrence. He passed away six months after treatment; however, this was secondary to pulmonary tuberculosis.

A case series of 215 pineal region tumors was published in the interim by a single surgeon between 1990 and 2017, of which 8 (3.7%) were glioblastoma [4]. The median age at diagnosis was 48.5 years, and 87.5% of patients were male. The most common symptoms were headache, vision changes, and gait imbalance/ataxia. The tumor origin for pineal region tumors was believed to be in the pineal gland in 3 (37.5%) of the cases with the others originating from the thalamus or indeterminate. The cause of symptoms was hydrocephalus, and it was managed by an endoscopic third ventriculostomy or ventriculoperitoneal shunt. In analyzing the eight patients whose glioblastoma was primary to or spread into the pineal gland, the median OS was 15 months, and tumors recurred locally except in one patient who had distal recurrence in the right frontal lobe. Recurrent subtotal resection was achieved in 75% of the eight-patient cohort, and all received standard fractionated external beam radiotherapy. One patient died perioperatively, and another was contraindicated for chemotherapy, while the rest were treated with TMZ as initial chemotherapy. Additional chemotherapies were attempted in 37.5% of the mixed group of eight patients. The perceived tumor origin did not influence the ability to achieve radical subtotal resection. Individual analysis of the three glioblastomas originating in the pineal gland was not reported.

The Güzel group described the case of a 5-year-old girl who was admitted with symptoms of headache, dizziness, difficulty walking, and impaired vision for one month [5]. A neurological exam showed sleepiness, unequal pupil size, inability to look laterally and weakness on the left side. An MRI revealed a mass in the pineal region that had spread to the right thalamus and superior peduncle and was determined to be a glioblastoma through histopathology. The patient had a shunt inserted for hydrocephalus, and the tumor was removed through a surgical approach. Treatment was still ongoing from seven months post-diagnosis.

A fourth case report detailed a 55-year-old female patient who was admitted to the hospital for dizziness, headache, blurred and double vision [6]. The physical examination revealed Parinaud syndrome, and an MRI confirmed a heterogeneously enhancing mass in the midline pineal region with ventriculomegaly. The patient underwent a surgical resection and a ventriculoperitoneal shunt to resolve the hydrocephalus. The histological diagnosis was glioblastoma. A genomic profiling showed telomerase reverse transcriptase (TERT) amplification, multiple TERT fusions, and FGFR2 fusions, as well as CDKN2A/CDKN2B loss, TP53 mutation, and 19q chromosome deletion. The patient received radiotherapy and TMZ chemotherapy, but her condition worsened leading to an overall survival of three months.

## Discussion

Glioblastoma is a highly malignant form of brain cancer with a poor prognosis. Glioblastoma of the pineal region is an extremely rare site, with 40 cases documented in the literature as of this review. The purpose of this manuscript, in addition to elaborating on an atypical case, is to summarize the current understanding of glioblastoma of the pineal gland through analysis of other case reports and their treatments. These reports have documented a variety of treatments for glioblastoma of the pineal region with an overall survival of less than three years. We previously reported the median survival of six months (range, 2-24 months) for pineal glioblastoma [1]. The available data in the cases published after 2017 [3-6] have similarly demonstrated a median survival of six months (range, 3-7 months). Excluding the present case, the median survival of all current cases in the literature stands at six months (range, 2-24 months).

In stark contrast to the median survival in other publications, the current patient showed a remarkable survival of 5.1 years where there was no evidence of progression of initial neurological deficits, and where the patient’s failure to thrive was possibly not secondary to his slowly progressive intracranial disease. The patient’s treatment differed in that the dose volume included a significant amount of the ventricular system, while other studies report irradiating the pineal region, but none have specifically mentioned expanding the volume into the ventricles to prevent leptomeningeal spread. In other tumors with common leptomeningeal spread, whole brain radiation therapy (WBRT) has been shown to improve survival [7-9]. Alternatively, whole ventricular irradiation has shown success in tumors such as germinomas in limiting disease to common areas of spread while maintaining reduced levels of cerebral toxicity and better cognitive function [10,11]. Our case employed this strategy to specifically irradiate the ventricular system with 50 Gy to prevent leptomeningeal spread due to proximity of the pineal gland.

Another distinction in the present case is the choice of systemic therapies. While a number of the published case reports utilized TMZ, the present case is the second case of pineal gland glioblastoma in the literature to receive bevacizumab as well [12]. In that case report, the addition of bevacizumab led to the treatment response of a pineal gland glioblastoma that was refractory to radiotherapy and TMZ. Although not initially involving the pineal gland, a recurrent glioblastoma case with leptomeningeal dissemination was also shown to have a clinical and radiographic response with the incorporation of bevacizumab therapy [13]. Part of the current patient’s longevity may also be in part due to the application of bevacizumab at initial recurrence, as a randomized trial of recurrent glioblastoma showed that those with an early response in the trial arm receiving bevacizumab had improved overall survival [14]. Ultimately, the irradiation of the ventricles when combined with bevacizumab at initial recurrence may explain the prolonged survival in our patient compared to the literature through the prevention and treatment of leptomeningeal spread.

Use of ChatGPT in assisting writing

ChatGPT is a state-of-the-art language model developed by OpenAI that uses deep learning algorithms to generate human-like text. It is trained on a massive amount of data, including a portion of medical literature, which makes it capable of generating coherent and informative responses related to medical topics.

One of the strengths of using ChatGPT for medical writing is its ability to quickly summarize input articles. This can be especially useful for medical professionals who need to quickly understand the main findings of a study or review a large number of articles in a short amount of time. Additionally, ChatGPT can be used to generate written content with remarkable speed, allowing medical professionals to focus their time and resources on more critical tasks. Quickly writing down the main topics the authors want to discuss, ChatGPT can quickly turn those points into an entire section of the paper. A summarized case by the authors can be used as input into ChatGPT, which outputs detailed and technical writing that surpasses the level of input, saving time for the authors in developing the language and summaries used in the analysis.

However, despite its training on medical literature, it is crucial to note that ChatGPT is not a substitute for professional medical advice. It is not capable of diagnosing or treating patients and should not be relied upon for making medical decisions. All medical advice generated by ChatGPT should be reviewed and approved by a licensed physician before it can be published or used in any official capacity. Another limitation of using ChatGPT for medical writing is that it has a knowledge cutoff of 2021, meaning it does not have access to more recent medical literature. Additionally, it cannot access the internet and is only able to generate responses based on the information it was trained on, so it may not be able to provide information on all medical topics or the most up-to-date findings for up-to-date literature reviews.

**Figure 3:**
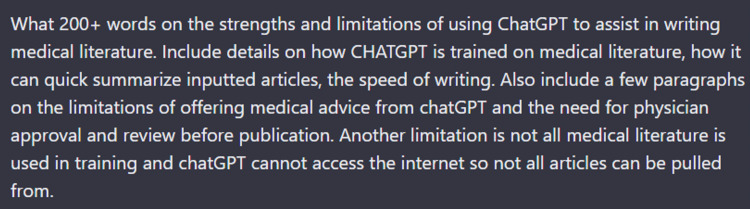
Screenshot of the ChatGPT query used to explain the strengths and weaknesses of ChatGPT use in medical literature. This query led to successfully synthesizing of concise conclusions with appropriate grammar. The generated output remains unedited in the first three paragraphs of the “Use of ChatGPT in assisting writing” section.

**Figure 4:**
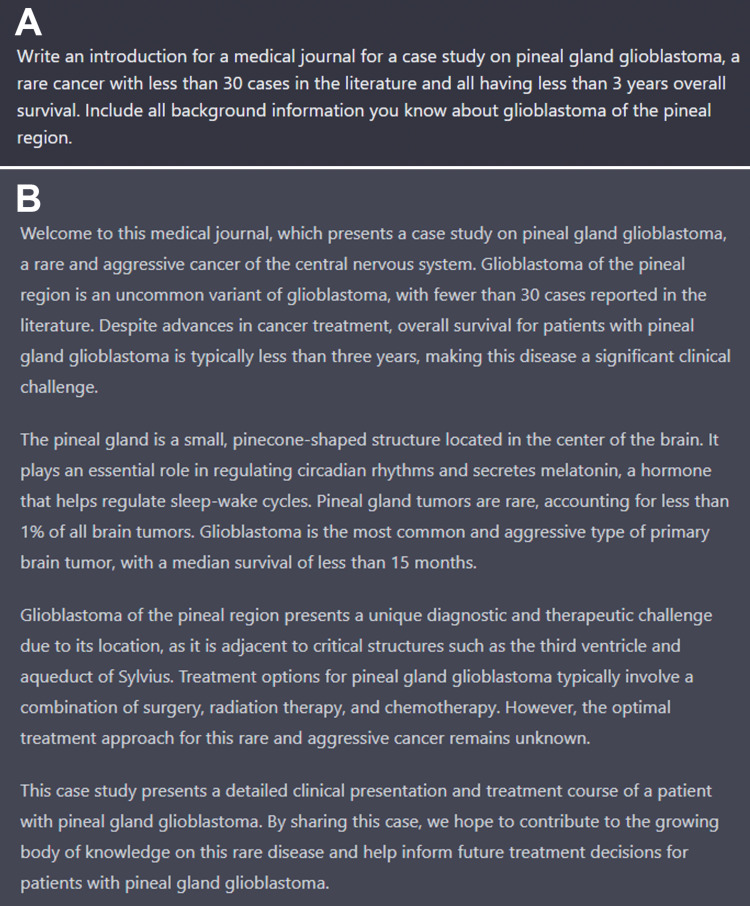
ChatGPT screenshot of input and output that developed the case report’s introduction. (A) Demonstrates the screenshot of the ChatGPT query used to generate the introduction, with the raw output shown in (B). While there were some modifications in the final draft of the manuscript, the current state of pineal glioblastoma in the literature was appropriately and concisely summarized with varied sentence structure.

With exception of the abstract and figure legends, all other parts of this article utilized ChatGPT to generate the foundation of the written text, which showed the strength in some tasks/aspects and weakness in others. For example, the contents of the prior three paragraphs are the successful unedited output from ChatGPT, with Figure 3 illustrating the input query that was used. Similarly, the general instruction given to ChatGPT in regard to creating the manuscript’s introduction rapidly created a well-detailed, comprehensive summary of pineal glioblastoma with syntax and overall structure that is hard to distinguish from human efforts (Figure 4). Updating some values and adding some missing content was all that was needed for the introduction as written.

**Figure 5:**
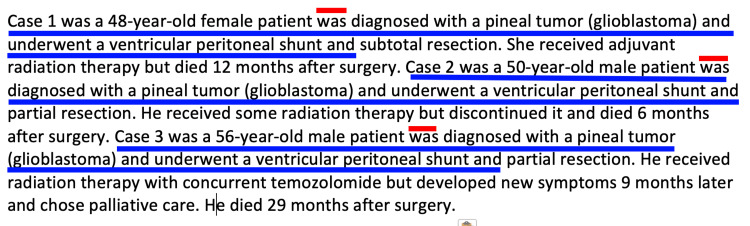
Example of ChatGPT leading to output with repetitive sentence structure and poor grammar. The ChatGPT instructions were to summarize the article by Orrego et al., with the entire contents of the manuscript entered into the input. As shown by the blue lines under the text, the output used the same exact sentence structure and wording to describe each case in the report. The red lines over the text highlights improper grammar, missing the identifier “who” before “was.”

**Figure 6:**
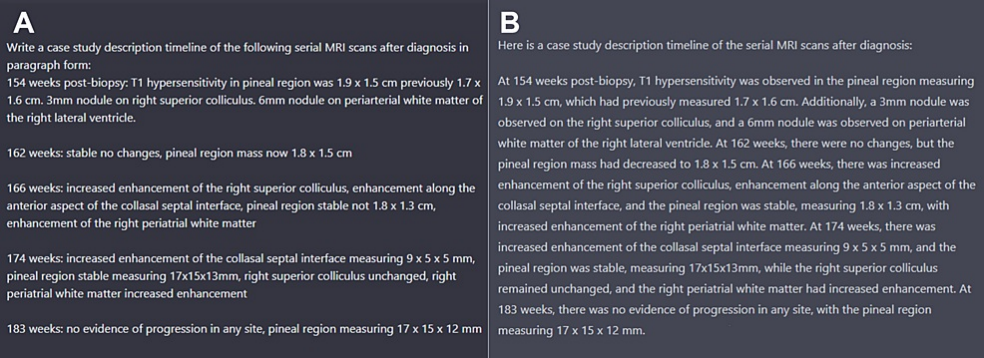
Screenshots of ChatGPT input and output were used to summarize findings in the case report’s patient follow-up. The query was to summarize the case report with the input being the image results by week post-biopsy as shown in (A). Figure (B) demonstrates the direct output from ChatGPT with very little fill between the input phrases and repetitive sentence structure.

In contrast to the two aforementioned tasks, we found that ChatGPT often produced paragraphs with style that was repetitive and grammar that was suboptimal. As exemplified in Figure 5, the summaries of the updated review of literature were prone to repetition of sentence structure, paragraph structure, and improper use of grammar. Artificial intelligence was able to understand the input of medical summaries or summarize a publication but lacked the ability to synthesize a complete picture of the patient's record and diagnosis. Further, there were some words that, to our knowledge, are not medical terms or jargon present in ChatGPT’s output, such as “mesh-like pupils.” In summarizing the patient's updated history, ChatGPT was able to take the rough MRI results and add some new details but failed to provide a stylistic summary or add any good overall insights into the progression of disease (Figure 6). Essentially, the language syntax in our generated responses had repeated syntax and sentence structure that required continued changes to become easily readable. While ChatGPT served as a time-saving aid in creating this manuscript, in most cases, it served as a rough draft that still required considerable human input to lead to an article with appropriate grammar, flow and content.

## Conclusions

In conclusion, glioblastoma of the pineal gland is a rare and challenging histology and site of brain cancer. Despite recent advances in treatments, the prognosis remains poor. Additional research into the addition of whole ventricular radiotherapy into the management of this site is warranted given this patient’s extended overall survival and lack of symptom progression. We believe that this volume strategy in preventing leptomeningeal spread likely contributed to this patient's atypical survival. Updating the review of literature for pineal glioblastomas further reinforced that the survival and treatment response on the present case is an outlier. In employing the assistance of ChatGPT in developing this manuscript, in most cases, the artificial intelligence platform did an acceptable job at creating a rough outline/draft, with greater success in developing particular parts of the manuscript. While ChatGPT can be a valuable tool for medical writing, it should be used with caution and in conjunction with professional medical advice and review. It is important to understand its strengths and limitations, and to always prioritize the health and well-being of patients.
